# Imaging Approach and Diagnosis in a Case of Combined Intraperitoneal: Extraperitoneal Urinary Bladder Injuries in a Child

**DOI:** 10.7759/cureus.96434

**Published:** 2025-11-09

**Authors:** Shabnam Bhandari Grover, Hemal Grover, Sanjay K Pal, Sumit Kumar, Ajay Sahni

**Affiliations:** 1 Radiology, School of Medical Sciences and Research, Sharda Hospital, Sharda University, Greater Noida, IND; 2 Radiology, Icahn School of Medicine at Mount Sinai West, New York, USA; 3 Pediatric Surgery, Maulana Azad Medical College (MAMC) and Lok Nayak Jayprakash (LNJP), New Delhi, IND

**Keywords:** cect combined with retrograde cystography, cect –delayed phase, combined intraperitoneal and extraperitoneal injuries, focused abdominal sonography, retrograde cystography, urinary bladder rupture

## Abstract

Urinary bladder rupture is a rare injury, both in adults and in children. Urinary bladder rupture in children is often due to road traffic trauma and is mostly of the intraperitoneal variety due to the higher anatomical location of the urinary bladder. Combined, post-traumatic, intraperitoneal, and extraperitoneal injuries, whether in adults or in children, are rare. The patient reported by us is a rare case of combined intra and extraperitoneal injuries in a four-year-old male child, without significant pelvic fractures. The boy, who sustained a road traffic accident a few hours earlier and was brought in a state of shock, with a history of anuria. Focused abdominal sonography for trauma (FAST), in the emergency room, was interpreted as “hemoperitoneum”. Adequate resuscitation and urinary bladder catheterization were followed by a contrast-enhanced CT scan (CECT). The delayed phase of the CECT scan at 30 and 45 minutes, without additional retrograde cystography, revealed the combined intraperitoneal-extraperitoneal injuries of the urinary bladder. Emergency surgery, with repair of the urinary bladder, led to a satisfactory recovery, and the boy was discharged in good condition at the end of three and a half weeks of hospitalization.

## Introduction

Urinary bladder rupture is a rare injury, which usually occurs due to blunt, motor vehicle-associated, abdominal or pelvic trauma; however, iatrogenic etiologies and spontaneous idiopathic rupture are also reported [1]. Intraperitoneal ruptures occur when a distended bladder is subjected to compressive forces over the lower abdomen; in this setting, the bladder gives way at the dome. Extraperitoneal injuries occur either due to compressive force on the lower abdomen, resulting in anterior or lateral bladder wall injury, or due to penetrating bone fragments from a pelvic girdle fracture, directly piercing the hollow viscus [1]. In adults, urinary bladder injuries occur in only 0.87-1.6% of all blunt abdominal trauma cases [2]. In the adult age group, 60% of traumatic bladder injuries are extraperitoneal, and these bladder injuries occur due to penetrating abdominal injuries or due to pelvic bone fractures in 89-100% of cases. The remainder are intraperitoneal, occurring due to a direct blow in the abdomen, with a distended bladder, with dehiscence at the bladder dome, which is the weakest part of the bladder. The latter mechanism is the etiology of seat belt-related trauma cases [2]. In the pediatric age group too, urinary bladder injuries are uncommon; these are also more frequently due to high-energy road-traffic-associated trauma and are of intraperitoneal variety due to the higher anatomical location of the urinary bladder in childhood [1,3,4]. The less frequently encountered aetiologies of urinary bladder injuries in the pediatric age group are iatrogenic injuries, trauma due to a fall from a height, cystography-related, and spontaneous or idiopathic rupture [5-11].

Combined, post-traumatic, intraperitoneal, and extraperitoneal injuries, whether in adults or in children, are rare. Grunherz et al. have described such an injury due to seat belt trauma in a 37-year-old driver, without pelvic fractures [2]. A similar, combined intra and extraperitoneal urinary bladder injury, without pelvic fractures, has been reported in a nine-year-old male child in a study by Singh et al. [12]. The patient reported by us is another rare case of combined intra and extraperitoneal injuries in a four-year-old male child, without significant pelvic fractures. The boy, who sustained a road traffic accident a few hours earlier, was brought to the emergency room, in a state of shock, with a history of anuria. Focused abdominal sonography for trauma (FAST) in the emergency room was interpreted as “hemoperitoneum”. Adequate resuscitation and urinary bladder catheterization were followed by a contrast-enhanced CT (CECT) scan. The delayed phase of the CECT scan at 30 and 45 minutes, without additional retrograde cystography, revealed the combined intraperitoneal-extraperitoneal injuries of the urinary bladder. However, despite the extraperitoneal leak, only a hairline, undisplaced fracture of the left pubic bone was documented. Emergency laparotomy with urinary bladder repair led to a satisfactory recovery, and the boy was discharged in good condition at the end of three and a half weeks of hospitalization. Most reports in the literature are focused on clinical features and surgical management protocols, whereas there is a relative paucity of reports emphasizing radiological techniques. Our report not only adds to the body of literature regarding a rare, combined intra and extraperitoneal bladder injury in a child, but also brings forth a slightly modified imaging technique and re-emphasizes the imaging appearances in this uncommon injury.

## Case presentation

A four-year-old male child who sustained a road traffic accident six hours earlier was brought to the emergency room around midnight, in a state of shock. There was a history of a single episode of painful, scanty micturition four hours prior, followed by anuria. Emergency room physical examination documented a drowsy child with lacerations on the left cheek, left hip, and pelvic bruising, associated with peritoneal guarding and rigidity. The Glasgow coma scale (GCS) was 15/15, blood pressure was 90/40 mmHg, tachycardia and tachypnea were recorded, oxygen saturation was, however, adequate, and the child was afebrile. Abdominal distension and guarding were both present, with bluish discoloration in the left flank. Standard procedure for resuscitation was instituted, which included intravenous fluids: adrenalin infusion, with eight-hourly broad-spectrum antibiotics, tetanus vaccination, and a nasogastric tube instillation-aspiration. Urinary bladder catheterization yielded no urine. Laboratory parameters showed a low hemoglobin of 7.5 gm /Dl, for which one unit of blood was transfused over a period of four hours. The prothrombin time was mildly prolonged, and the international normalized ratio (INR) was slightly raised. FAST in the emergency room was interpreted as “hemoperitoneum”. The surgical team suspected a urinary bladder injury, and as soon as the blood pressure stabilized, a CECT scan was requisitioned in the early morning hours. The study was conducted using all standard precautions to minimize radiation dose.

**Figure 1:**
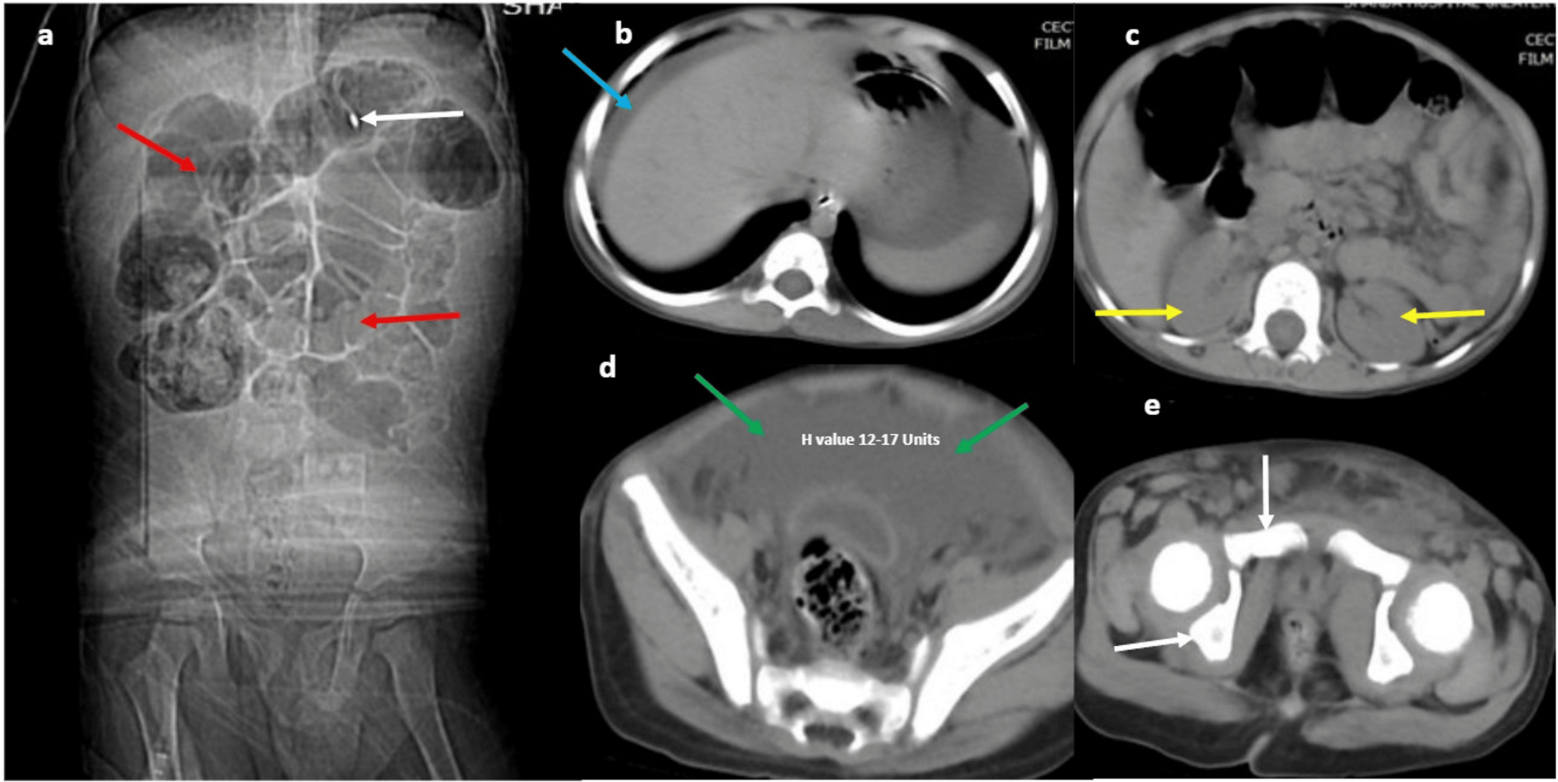
a-e: NCCT scan images of the abdomen and pelvis in a four-year-old male child with a very recent road traffic injury followed by abdominal pain, distension, anuria, and suspected urinary bladder injury. The scout view (a) shows the nasograstic tube in situ (white arrow), with diffusely dilated small and large bowel loops (red arrows in a). The upper abdomen (b) shows free peritoneal fluid around the hepatic capsule (blue arrow in b). Both kidneys and perirenal tissues appear normal (yellow arrows in c). Extensive free fluid, with attenuation of 12-17 HU, is seen in the pelvis (green arrows in d). The pubic bones and iliac bones (white arrows in e) appear normal. NCCT: non-contrast computed tomography; HU: Hounsfield Unit

The CT (scout view) showed paralytic ileus (Figure 1a). The non-contrast computed tomography (NCCT) phase showed free peritoneal fluid in the upper and lower abdomen and pelvic regions; the pelvic bone appeared normal in the standard abdomen windows (Figures 1b-1d). The renal outlines and peri-renal tissues appeared normal (Figures 1b-1d).

**Figure 2:**
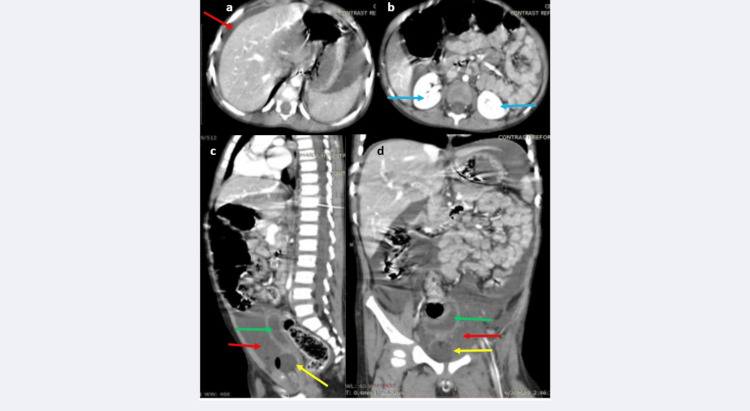
a-d: CECT scan (early phase, 60 seconds) shows the liver and spleen are intact with normal enhancement and normal margins, free peritoneal fluid is present (red arrow in a). Both kidneys show prompt and good enhancement without any perirenal leaks (blue arrows in b). The pelvic cavity reveals free peritoneal fluid, without any contrast staining (red arrows in c, d). The urinary bladder appears collapsed (green arrows in c, d). Foley's catheter is seen at the urinary bladder neck region (yellow arrows in c, d). CECT: contrast-enhanced CT scan

The contrast-phase CT showed a normal liver, spleen, and bilateral, promptly excreting kidneys with a normally enhancing aorta. The free peritoneal fluid did not show any contrast enhancement or leak into the peritoneal cavity in the early phase (Figures 2a-2d).

**Figure 3:**
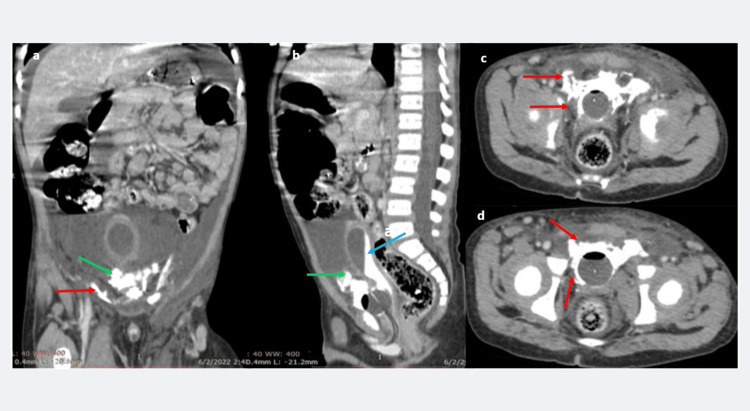
a-d: CECT scan (delayed phase, 30 minutes) images reveal, extravasation of contrast around the anterior bladder wall into the pelvic peritoneum (green arrow in a, b), a contracted and poorly distending urinary bladder is seen (blue arrow in b). Additionally, extensive extravasation of contrast is seen into the extraperitoneal, extra-vesical space (red arrows in a, c, d). This phase of the study demonstrates the combined intraperitoneal-extraperitoneal urinary bladder injury. CECT: contrast-enhanced CT scan

The urinary bladder was seen with a Foley’s catheter in situ (Figures 2c-2d). The scan obtained at 30 minutes showed a partially contrast opacified and contracted urinary bladder with disruption along its anterior-inferior wall, leading to active and extensive contrast extravasation into the peritoneal cavity (Figures 3a-3b). In addition, contrast extravasation was seen around the Foley’s catheter and urinary bladder neck region (Figures 3c-3d).

**Figure 4:**
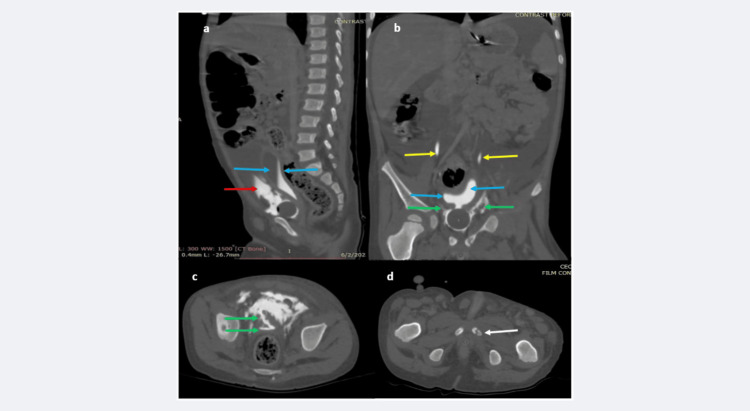
a-d: CECT scan (delayed phase, 45 minutes), bone window-viewed scans, reveals extravasation of contrast around the anterior bladder wall into the pelvic peritoneum (red arrows in a), the urinary bladder is seen as poorly distending (blue arrow in b, c). The ureters are normal (yellow arrow in b); however, extravasation of contrast is seen all around the bladder neck, into the perivesical space (green arrows in b, c). A hairline fracture is seen in the left pubic bone (white arrow in d). This phase of the study further reconfirms the combined intraperitoneal-extraperitoneal urinary bladder injury and demonstrates the insignificant, hairline fracture in the left pubic bone. CECT: contrast-enhanced CT scan

The CT scan at 45 minutes, viewed at the bone window, documented the anterior inferior wall bladder disruption with intraperitoneal contrast extravasation, with an additional urinary bladder-neck region and prostatic-urethra disruption with extraperitoneal contrast extravasation (Figure 4a-4c). The ureters appeared normal (Figure 4c), and a hairline, undisplaced left pubic bone fracture was documented (Figure 4d).

**Figure 5:**
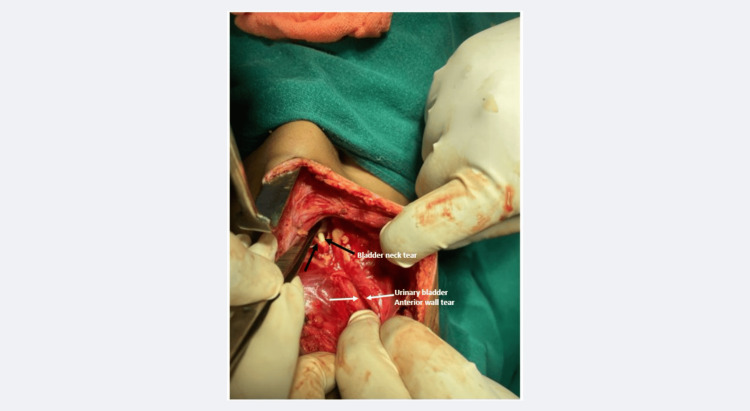
The intraoperative photograph at the initial stage shows the large, anterior bladder wall tear (white arrows) and the additional tear at the bladder neck (black arrows).

The patient was shifted to the operating theatre, and a lower midline incision showed that the entire peritoneal cavity was filled with blood mixed with urine. Peritoneal lavage revealed a large tear in the anterior bladder wall with another injury extending from the urinary bladder neck to the prostatic urethra (Figure 5).

**Figure 6:**
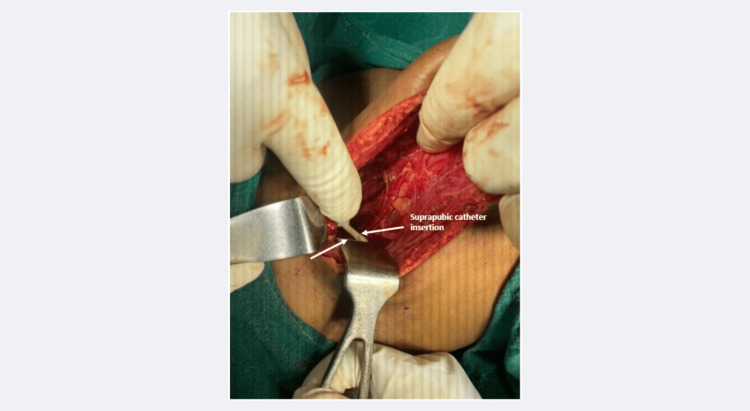
The intraoperative photograph at the post-repair stage shows the suprapubic intravesical catheter being placed in situ (white arrows).

The Foley's catheter was seen through the bladder neck injury (Figure 5). The urinary bladder anterior wall was repaired in two layers, and a suprapubic catheter was placed in the bladder lumen (Figure 6).

The bladder neck and prostatic urethra were also repaired in two layers around the Foley's catheter. Additionally, a retro-pubic, pre-vesical-space pelvic drain was placed in situ. The abdomen was closed in layers. The boy had an uneventful recovery, and the retro-pubic drain was removed on day 7 of surgery, as there was negligible drainage. The suprapubic urinary catheter was clamped on day 10, and adequate urine output from the trans-urethral Foley’s catheter was documented. The transurethral Foley's catheter was removed on day 17, and the suprapubic catheter was removed on day 20. Subsequently, the patient demonstrated normal urethral voiding, all clinical and laboratory parameters were normal, and the boy was discharged on the twenty-third day. Follow-up outpatient visits at three months and six months were satisfactory.

## Discussion

The urinary bladder remains fixed in its deep pelvic location by reflections of the visceral pelvic fascia, further reinforced by true and false pelvic ligaments, pubo-prostatic ligaments (in males), and the urogenital diaphragm, which stabilize the bladder neck. In addition to the ligamentous structures, the urinary bladder neck is held in position by the obturator internus muscle. The peritoneal reflection in adults lies over the posterosuperior surface of the urinary bladder. The anterior compartment between the anterior abdominal wall and the anterior wall of the urinary bladder is supported by connective tissue [12]. During childhood, the urinary bladder is anatomically in a higher location and descends to the pelvic location only by the age of 20 years [12].

The American Association for the Surgery of Trauma (AAST), classifies urinary bladder injuries into five grades of severity: grade 1 is a contusion or intramural hematoma; grade 2 is extraperitoneal rupture, with a wall laceration <2 cm size; extraperitoneal rupture >2 cm or intraperitoneal <2 cm bladder wall laceration comprise a grade 3 injury; intraperitoneal bladder wall lacerations >2 cm are grade 4 injuries, while an injury at the urinary bladder neck, whether communicating with intraperitoneal or extra peritoneal compartment, is categorized as a grade 5 injury [13]. Pediatric surgeons, however, classify urinary bladder injuries as four types: bladder contusion, extra-peritoneal rupture, intraperitoneal rupture, and a combined variety [4]. The patient described by us had a combined intraperitoneal and extraperitoneal injury.

The clinical symptoms and signs in a post-traumatic, intraperitoneal insult to the urinary bladder are abdominal bruising (seat belt sign), abdominal/pelvic pain, tenderness, distension, dysuria, haematuria, inability to urinate, anuria, or absence of urinary output through a catheter [1,2,4,5]. In the event of a delayed diagnosis, resorption of the intraperitoneal urine may lead to life-threatening electrolyte and metabolic derangements [1,3]. Extraperitoneal bladder injury may present with similar symptoms of pelvic bruising, pelvic pain, dysuria, haematuria, inability to urinate, or absence of urinary output through a catheter. Additionally, extraperitoneal leak should be suspected in patients with overt pelvic bone fractures and patients who have urinary extravasation into the anterior abdominal wall, thigh, or scrotum [12]. Injuries involving the pubic rami are more likely to cause anterior wall injury, while those through the sacrum are associated with posterior bladder wall injuries [1,12]. The reported case showed the classical manifestations of an intraperitoneal bladder injury. It was the delayed phase of a CECT study that demonstrated the additional, co-existing extraperitoneal injury, with contrast extravasation around the bladder neck and prostatic urethra. A hairline, non-displaced left pubic bone fracture was the only bone injury documented in our patient.

The unusual observation in our patient was the conspicuous absence of significant pelvic fractures, which could explain the extraperitoneal injury. Singh et al. have described a similar case in a male child with trauma and extraperitoneal leak. These authors have hypothesized that severe shearing force injuries may disrupt the bladder neck, even in the absence of penetrating bone fragments, and result in an extraperitoneal variety of bladder rupture [12]. A similar mechanism was likely in our patient, who was the victim of a high-energy, blunt abdomino-pelvic vehicular accident.

The primary investigation in a trauma patient with abdominal pain and distension remains a FAST examination to document or rule out hemoperitoneum. The specific recommended diagnostic investigation in urinary bladder trauma was to perform a conventional cystography, which has now been majorly replaced by CECT combined with active retrograde cystography [1,2,4,7,13 ]. However, we used a modified approach, utilizing the delayed phase of CECT (30 minutes and 45 minutes) as the passive cystography phase, thus avoiding further intervention with an additional retrograde cystogram. The technique adopted by us provided all the necessary information, without further intervention and distress to the young child. The same technique will work equally well in patients with an additional urethral injury, where it might not be technically feasible in a retrograde catheterization. This technique is further relevant in the light of a few case reports of bladder rupture in children attributed to voiding cystography itself [8,9]. The CECT study protocol described in our patient thus underscores the importance of delayed phase scan acquisitions and review in cases suspected of having urinary bladder perforation, without additional retrograde cystography. The described approach also emphasizes the importance of bone window viewing in all cases of trauma.

Recommendations for the management of an intraperitoneal urinary bladder injury are surgical intervention and bladder wall repair, supported by suprapubic and transurethral catheter drainage for two to three weeks [13,14]. The stepwise, gradual withdrawal of drainage catheters is an additional vital protocol. A similar strategy of surgical repair with combined suprapubic-transurethral catheter drainage, followed by a regimented protocol of catheter withdrawal, was followed in our patient, with a satisfactory outcome. In patients with isolated and uncomplicated extraperitoneal injuries, the recommended treatment is a conservative approach, with catheter drainage. However, complex extraperitoneal and penetrating injuries should undergo surgical repair [13,14].

## Conclusions

Although injuries to the urinary bladder are rare in cases of accidental trauma in both adults and children, the latter are more prone due to a relatively higher anatomical location of the urinary bladder during early life. The adult bladder, lying deep within the pelvic cavity and robustly anchored, is less frequently injured, unless it suffers a direct abdominal force in a distended state. During childhood, the bladder, being higher in anatomical location, is more likely to suffer from an intraperitoneal injury, as compared to adults. Extraperitoneal bladder injuries may occur in adults or in children due to severe shearing forces or due to penetrating pelvic bone fractures. A combined variety of injuries, as seen in our patient, remains rare, both in adults as well as in children. The standard investigation technique for suspected urinary bladder injury is a CECT scan combined with retrograde cystography. However, we have demonstrated that a delayed phase of CECT, at 20-30 minutes, which is the bladder filling phase, is equally effective for demonstrating injuries. The described CECT technique without retrograde cystography is important for obviating further intervention and further risk of injury, as explained above. Our report additionally highlights the importance of bone window views in trauma patients. The standard recommendations are in favor of surgical repair and supported bladder drainage, for intraperitoneal and for complex extraperitoneal injuries, with simple catheter drainage recommended only for uncomplicated extraperitoneal injuries.
